# Targeting sterol-O-acyltransferase 1 to disrupt cholesterol metabolism for cancer therapy

**DOI:** 10.3389/fonc.2023.1197502

**Published:** 2023-06-20

**Authors:** Teng Tu, Hongying Zhang, Huanji Xu

**Affiliations:** ^1^ Department of Medical Oncology, Cancer Center and Laboratory of Molecular Targeted Therapy in Oncology, West China Hospital, Sichuan University, Chengdu, China; ^2^ Laboratory of Oncogene, West China Hospital, Sichuan University, Chengdu, China

**Keywords:** sterol-O-acyltransferase 1, cholesterol metabolism, cancer therapy, avasimibe, cholesterol esterification

## Abstract

Cholesterol esterification is often dysregulated in cancer. Sterol O-acyl-transferase 1 (SOAT1) plays an important role in maintaining cellular cholesterol homeostasis by catalyzing the formation of cholesterol esters from cholesterol and long-chain fatty acids in cells. Many studies have implicated that SOAT1 plays a vital role in cancer initiation and progression and is an attractive target for novel anticancer therapy. In this review, we provide an overview of the mechanism and regulation of SOAT1 in cancer and summarize the updates of anticancer therapy targeting SOAT1.

## Introduction

Sterol O-acyl-transferase 1 (SOAT1), also known as acyl-CoA:cholesterol acyltransferase 1 (ACAT1), is an exclusively intracellular enzyme that catalyzes the formation of cholesterol esters from cholesterol and long-chain fatty acids in cells. It plays an important role in maintaining cellular cholesterol homeostasis (1–3). In the early time, SOAT1 has been studied extensively as a potential drug target in atherosclerosis and Alzheimer’s disease (4, 5). In recent years, many studies have found that tumors such as glioblastoma and pancreatic cancer exhibit high expression of SOAT1 accompanied by high cholesteryl esters, indicating that SOAT1 is also crucial for cancer cells to regulate cholesterol metabolic homeostasis (6). Compelling evidence have implicated that targeting SOAT1 is a promising therapeutic strategy for cancer management. In this review, we mainly discuss the cholesterol metabolism centered on SOAT1 and mechanisms that regulate SOAT1 expression in cancers. Finally, the latest developments that target SOAT1 for cancer treatment are summarized.

## Cholesterol metabolism centered on SOAT1

**Figure 1:**
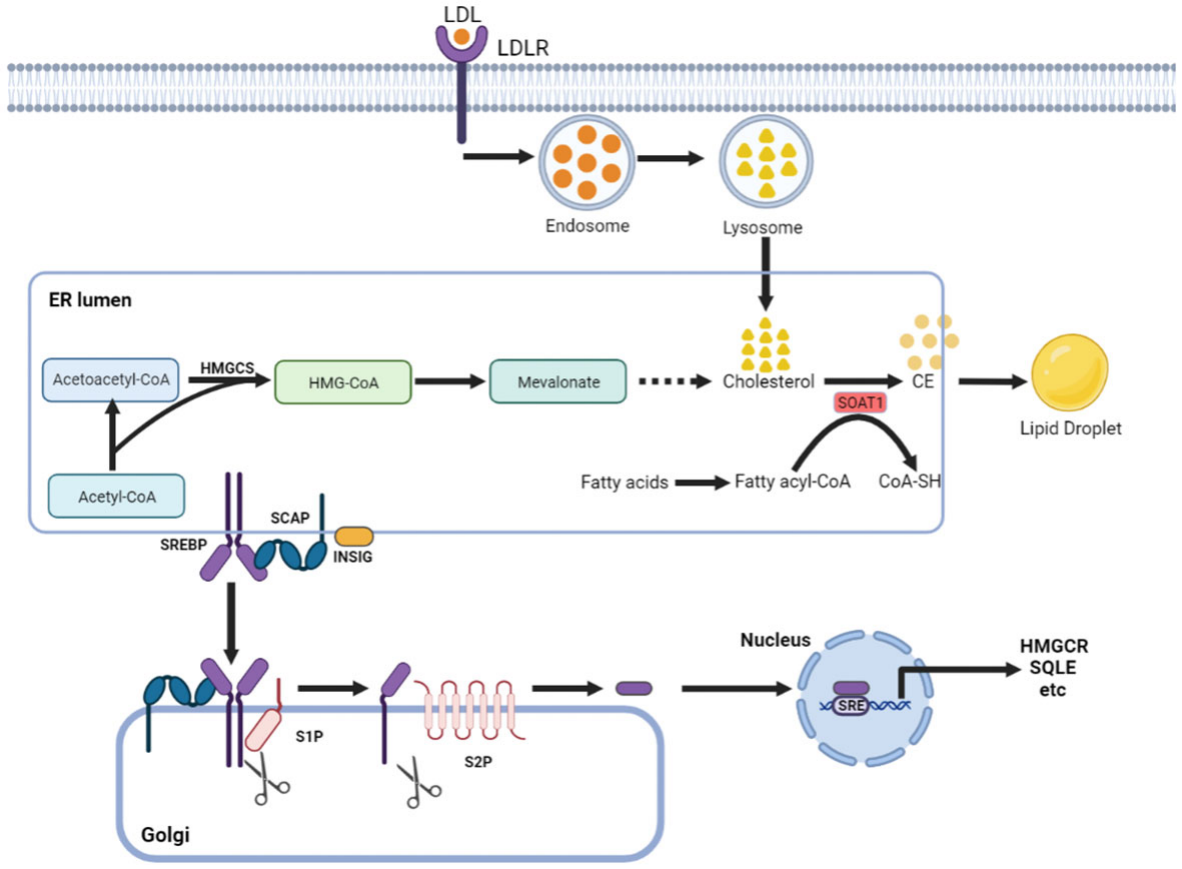
Cholesterol metabolism centered on SOAT1. Cholesterol acquisition by cells occurs through *de novo* synthesis in the endoplasmic reticulum (ER) and via LDLR-mediated endocytosis of LDL. SOAT1 is an ER-associated enzyme responsible for cholesterol storage. SOAT1-mediated esterification of increased cholesterol and fatty acids results in the formation of CE, which is stored in lipid droplets. SREBP cleavage-activating protein (SCAP) is a cholesterol sensing protein and forms a complex with SREBP2 and an ER membrane anchor protein insulin-induced gene (INSIG). A decrease in cholesterol levels within the endoplasmic reticulum (ER) results in the dissociation of INSIG from SCAP, thereby releasing the SCAP/SREBP2 complex. Subsequently, SREBP2 is transported to the Golgi complex and undergoes proteolytic activation by the site-1 and site-2 proteases (S1P and S2P). The N-terminal domain of SREBP2 then translocates to the nucleus to initiate gene transcription necessary for cholesterol synthesis and uptake.

Cholesterol can maintain membrane fluidity and integrity and form membrane microstructures as an essential lipid component of the mammalian cell membrane (7). Intracellular cholesterol metabolic homeostasis is determined by a complex network that regulates cholesterol biosynthesis, uptake, export, and esterification (**Figure 1**) (8). Almost all mammalian cells can *de novo* synthesize cholesterol from acetyl-CoA to cholesterol through more than 20 enzymatic reactions. Besides that way, most cells absorb cholesterol from low-density lipoprotein (LDL) via LDLR-mediated endocytosis (9), where the complex is internalized into the cell endosome by endocytosis with the binding of LDL to LDLR. In the endosome, LDL is dissociated from LDLR and further transferred to the lysosome, where the free cholesterol is released from LDL.

Increased cholesterol can be converted to cholesteryl ester (CE) by sterol O-acyltransferase (SOAT) and stored in lipid droplets (10). There are two genes encoding the two SOAT enzymes, SOAT1 and SOAT2 (11). SOAT1 is widely expressed in most cell types and highly expressed in some tumors (12). SOAT2 is mainly distributed in hepatocytes and intestinal epithelial cells (13). Despite being essential for cell viability, cholesterol is not always beneficial; in contrast, when in excess, it is highly toxic, and its levels must therefore be tightly controlled. This is, in part, achieved by SOAT1-mediated cholesterol esterification (14). Sterol regulatory element-binding proteins (SREBPs) are core transcription factors that can sense cholesterol content in endoplasmic reticulum (ER) membrane and control the expression of important genes in cholesterol synthesis and uptake (15, 16). Elevated cholesterol content can inhibit the activity of SREBPs. SOAT1 can alleviate the inhibitory effect of SREBPs activity by converting cholesterol into CE (17), thus promoting the synthesis and absorption of cholesterol.

The precise control of free cholesterol content makes SOAT1 as a key intrinsic driver of cholesterol metabolism. Previous studies showed that SOAT1 plays a crucial role in the accumulation of foam cells from macrophages, the key pathological process in atherosclerosis. Besides, SOAT1 can promote the formation of very low-density lipoprotein (VLDL) in the liver, and the absorption of dietary cholesterol from the intestines (18). Targeting SOAT can directly lower the level of plasma cholesterol and inhibit the formation of arterial plaque, which may significantly reduce the risk of atherosclerosis and other cardiovascular diseases, although relevant phase III clinical trials were failed (19). Cholesterol metabolism is also closely associated with Alzheimer’s disease at several stages (5). Cholesterol levels affect the processing of amyloid protein and its precursors (20). Genetic knockout or pharmacological inhibition of SOAT1 have also been proved to provide several beneficial effects on Alzheimer’s disease.

## Clinical relevance of SOAT1 in cancer

In recent years, several studies have indicated that cholesterol esterification is deregulated in cancers, and SOAT1 has received increasing attention for its association with cancer (17). SOAT1 is widely expressed across various cancers, such as breast cancer (21), renal cancer (22), liver cancer (12), glioma cancer (23), pancreatic cancer (24) and adrenocortical cancer (25). SOAT1 can play an essential role in cancer cell proliferation, migration, invasion and metastasis (26). As reported in previous studies, deregulated SOAT1 is associated with tumor aggressiveness and therapy resistance, indicating poor prognosis in different kinds of cancers (17).

Typically, high expression of SOAT1 is a specific signature of hepatocellular carcinoma (HCC), which can regulate the distribution of cellular cholesterol and promote HCC cell proliferation (27). Meanwhile, SOAT1 knockdown suppresses cell proliferation and migration in HCC (12, 28). In gastric cancer (GC), overexpression of SOAT1 could promote cholesterol ester synthesis and GC cell proliferation. Meanwhile, SOAT1 can promote gastric cancer lymph node metastasis via regulating lipid synthesis (29). Mechanistically, SOAT1 can upregulate the activities of SREBPs, which induces lymphangiogenesis by increasing the expression of VEGF-C.

In addition, high SOAT1 expression is also associated with lymph node metastasis, which indicates poor patient disease-free survival and overall survival in colorectal cancer (CRC) (30, 31). In prostate cancer, high expression of SOAT1 upregulates the expression levels of SREBPs and LDLR and eventually promotes cancer proliferation (32). A recent study found a significantly shorter median biochemical recurrence (BCR)-free survival of 93 months in patients with high SOAT1 compared to 134 months with low SOAT1 (33). In HCC, the protein expression of SOAT1 was significantly increased in the tumor compared with adjacent tissue (34). A proteomics study found that HCC patients with disrupted cholesterol metabolism and high expression of SOAT1 tend to have a poorer prognosis (12). Collectively, these results show that high expression of SOAT1 is associated with poor prognosis and promotes the growth and migration of different kinds of tumors.

## Mechanisms that regulate SOAT expression in cancer

SOAT1, as the main enzyme that converts cholesterol into CE, is regulated exquisitely by a complex network, including the transcription program and the posttranscriptional program. Recent studies have reported some novel mechanisms that result in the high expression of SOAT in cancer.

## Transcriptional regulation

Previous studies have found that the SOAT1 promoter can be activated by IFNγ, TNF, insulin and glucocorticoids (14, 35, 36). However, no binding site for the classic cholesterol metabolism transcription factors SREBPs or LXRs has been identified in the SOAT1 promoter (14). Recent studies have shown that the mRNA levels of SOAT1 are also directly regulated by P53, β-catenin and piRNA-6426.

P53, the tumor suppressor, plays a central role in cancer development. Over 60% of human cancers carry *P53* gene missense mutations or deletions (37). P53 has the ability to repress cholesterol biosynthesis and restrict tumor growth by blocking cholesterol efflux (38). The recent study also indicated that P53 can directly bind to the promoters of SOAT1 genes and lead to its transcriptional attenuation (39). The oncogene β-catenin can also regulate cholesterol esterification by directly activating the transcriptional expression of SOAT1 (30). piRNAs are small RNAs with a length of approximately 30 nt that can guide PIWI proteins to silence transposable factors by binding to DNA methyltransferases (40). The overexpression of piRNA-6426 could increase the methylation level of the SOAT1 promoter, which can reduce the expression level of SOAT1 mRNA and protein, while the interference of piRNA-6426 can enhance the expression levels of SOAT1 mRNA and protein (41).

## Posttranscriptional regulation

SOAT1 is regulated not only by the transcription program but also by the posttranscriptional program. Studies have shown that P53 can not only directly regulate the mRNA levels of SOAT1 involved in cholesterol metabolism but can also regulate the protein levels of SOAT1 by regulating the mRNA levels of USP19 (39). Ubiquitin-specific peptidase 19 (USP19) is a deubiquitinating enzyme (DUB) that stabilizes SOAT1 by decreasing its ubiquitination (42). Potential ubiquitination of SOAT1 includes the ϵ-amino groups of the protein’s seven lysine residues (K6, K11, K27, K29, K33, K48, and K63). USP19 can stabilize SOAT1 by decreasing K33/K48-linked ubiquitination (38). In addition, a previous study have also reported that SOAT2, the homologous protein of SOAT1 can be regulated by cholesterol and fatty acids (FAs). High levels of cholesterol and FA can prevent SOAT2 from ubiquitination on C277 cite and degradation via inducing ROS (43). However, whether cholesterol and FA have the same effect on SOAT1 is still unknown.

## Tumor promotion by high SOAT1 expression

Cholesterol metabolism has been considered to play essential roles in tumor growth and metastasis (17). As the key enzyme in the steps of cholesteryl ester synthesis, the activity of SOAT determines the abundance of cholesterol in cancer. The excess free cholesterol, together with the fatty acyl CoA substrate can be converted to CE by SOAT1 and stored in LDs. Some studies also emphasized that it is the role of cholesteryl ester rather than cholesterol that promotes tumor growth (44–47). Given that cholesterol and fatty acids are important components of the cell membrane, cancer cells may use these stored CEs for new cell membranes and signaling molecules, which can promote the growth of tumors. Studies have showed that high SOAT1 expression elevated lipid availability, which can promote tumor aggressiveness (12, 32).

There are three SREBP isoforms. SREBP-1a and SREBP-1c mainly regulate fatty acid synthesis, and SREBP-2 controls cholesterol synthesis (48, 49). They all have the function of controlling lipid and cholesterol homeostasis (50). Normally, SREBP activity is tightly regulated by negative feedback, which is controlled by endoplasmic reticulum (ER) membrane cholesterol (14). However, studies found that tumor cells could evade high levels of cholesterol-induced negative feedback inhibition as well as maintain SREBP activity (51, 52). In PTEN-deficient tumors, the PI3K/AKT/mTOR pathway is activated, which in turn upregulates SREBPs-mediated cholesterol synthesis and absorption. However, elevated cholesterol content can inhibit the activity of SREBPs. SOAT1 could alleviate the inhibitory effects of SREBPs via converting cholesterol into CE, thus promoting the growth of PTEN-deficient tumors. The deficient of SOAT1 or SOAT1 inhibition would disturb cholesterol homeostasis, and then inhibits the activities of SREBPs and eventually suppresses tumor development (32).

In addition, SOAT1 can suppress the function of CD8^+^ T cells. CD8^+^ T cells have a central role in antitumor immunity. However, numerous studies have shown that their activity is suppressed in the tumor microenvironment (TME) (53–56). Inhibiting cholesterol esterification in T cells by SOAT1 inhibitors can enhance the proliferation of CD8^+^ T cells and their effector function. This is because when the cholesterol level of CD8^+^ T cells decreases, T-cell receptor clustering and signaling as well as efficient formation of the immunological synapse were suppressed (57). SOAT1 expression in tumors might also affect immune infiltration in the TME. A recent study noted that SOAT1 expression was positively correlated with various tumor infiltrating immune cells in glioma. More importantly, SOAT1 expression was found to be positively correlated with multiple chemokine/chemokine receptor gene and various checkpoint genes, including PD-L1. These results indicated that SOAT1 overexpression may affect immune infiltration and tumor immune escape via increasing the secretion of chemokines and the levels of checkpoint genes (58). Nevertheless, both the mechanism by which SOAT1 promotes tumor growth and immune escape need to be further studied.

## SOAT1-targeted therapeutic strategies for cancer treatment

The process by which SOAT1 converts cholesterol into CE occurs in many types of aggressive cancer. High SOAT1 expression in tumors has been considered to be associated with higher grades of breast and renal cancer, respectively (21, 22). In addition, high expression of SOAT1 has also been considered to be associated with poor prognosis for patients with liver (12), glioma (23), pancreatic (24) and adrenocortical cancers (25). Thus, SOAT1 inhibition could be pursued as a therapeutic strategy for cancers (**Table 1**).

**Table 1:** SOAT1-targeted therapies in cancer.

Drug	Target	Cancer type	Phase	Mechanism	Reference
Avasimibe	SOAT1/2	Prostate cancer and glioblastoma	*In vivo* preclinical experiments	Inhibits the activities of SREBPs and eventually suppressing cancer proliferation	(32, 44)
		Pancreatic cancer	*In vivo* preclinical experiments	Promotes cholesterol-induced ROS and ER stress	(59)
		HCC	*In vivo* preclinical experiments	Disrupts cholesterol metabolism homeostasis and decreases cholesterol esterification	(12, 39)
		Gastric cancer	*In vitro* experiments	Suppresses GC cell proliferation, cholesterol ester synthesis, and lymphangiogenesis	(29)
ATR-101 (Nevanimibe)	SOAT1/2	Adrenocortical carcinoma	In phase I clinical trials	Suppresses adrenal steroidogenesis at lower doses and causes apoptosis of adrenocortical cells at higher doses	(60)
Nilotinib	SOAT1	HCC	*In vivo* preclinical experiments	Displays a high effect on SOAT1 protein and significantly inhibit tumor activity both *in vitro* and *in vivo*	(61)

Avasimibe, first discovered in 1996, is the first and most frequently used SOAT1 inhibitor (62, 63). It has been clinically verified to be safe and effective for cancer treatment in preclinical studies (64). Therefore, targeting SOAT1 with avasimibe may be a safe and effective method to disrupt cholesterol metabolic homeostasis in cancer treatment. In prostate cancer and glioblastoma, avasimibe could increase intracellular cholesterol levels, thus inhibiting the activities of SREBPs and eventually suppressing cancer proliferation (32, 44). In pancreatic cancer, avasimibe can promote tumor cell apoptosis by increasing intracellular cholesterol-induced ER stress (59). In HCC, SOAT1 inhibition by avasimibe dramatically repressed high-cholesterol high-fat diet (HCHFD)-induced HCC tumorigenesis and decreased cholesterol esterification, which markedly reduced tumor growth in HCC with high SOAT1 expression (12, 39). In gastric cancer, the inhibition of SOAT1 by avasimibe could suppress GC cell proliferation, cholesterol ester synthesis, and lymphangiogenesis (29). In addition, SOAT deficiency in cytotoxic T lymphocyte (CTL) leads to enhanced T-cell receptor clustering and increased cholesterol content in the plasma membrane, which can enhance CTL cytotoxicity, and synergize with immunotherapy in controlling cancer development and growth (57, 65, 66).

In addition to avasimibe, there are also some other compounds that can suppress SOAT1 activity. ATR-101 (nevanimibe) is a SOAT1-specific inhibitor that has been tested against adrenocortical carcinoma in clinical trial. This study demonstrated the safety of nevanimibe, but with limited efficacy in patients with advanced ACC (60). A recent study also screened three compounds, nilotinib, ABT-737, and evacetrapib, that exhibited optimal binding with SOAT1 and inhibitive activities. In particular, nilotinib, a second-generation tyrosine kinase inhibitor used in the clinic, displayed a high affinity for SOAT1 protein and significantly inhibited tumor activity both *in vitro* and *in vivo* by reprogramming the intracellular cholesterol metabolism of tumor cells and enhancing the effect of CD8+ T cells and neutrophils (61).

In addition, the latest research has also explored combination strategies based on avasimibe for cancer treatment. A previous study showed that simultaneously targeting SOAT1 and CPT1A by avasimibe and etomoxir had cooperative anticancer efficacy in HCC *in vitro* and *in vivo* via increasing the intracellular levels of cholesterol and fatty acids (28). Nystatin, a polyene antifungal drug for managing cutaneous or mucosal candidiasis, can synergize with avasimibe in suppressing the viability of colon cancer cells *in vitro* and *in vivo* via cholesterol sequestration and decreasing cholesterol-promoted oncogenic signals (67).

## Conclusion

SOAT1 plays an important role in maintaining cellular cholesterol homeostasis by meeting the requirement of cancer cells for cholesterol. The high abundance of SOAT1 in tumors indicates worse prognosis. Therefore, SOAT1 seems to be an attractive target for novel anticancer therapy. It is glad that an increasing number of preclinical studies have revealed the antitumor effects of SOAT1 inhibition and relative mechanisms. Several SOAT1 inhibitors, such as avasimibe and nevanimibe, have shown a good human safety profile in clinical trials, making them promising approach to fight against tumors. However, these clinical trials have proven to be ineffective in retarding atherosclerosis (68, 69), probably due to the nonspecific binding and low absorptivity. To date, clinical trials have not extensively evaluated the efficacy of SOAT1 inhibitors for cancer treatment, with the exception of nevanimibe in a phase I study (60). To facilitate their clinical implementation, it is imperative to conduct large-scale clinical trials and explore combination therapy. The elucidation of the SOAT1 protein crystal structure provides an opportunity to develop a more potent and safer SOAT1 inhibitor with specific binding and high absorptivity, potentially paving the way for the next generation of SOAT1-targeted therapy in cancer (70).

## Author contributions

HX made contributions to the conception and design. TT and HX wrote the manuscript. HZ helped to revise the manuscript and collect the data. All authors contributed to the article and approved the submitted version.
